# Characterizing individual differences in children and adolescents with autism spectrum disorder: a descriptive study

**DOI:** 10.3389/fpsyg.2024.1323787

**Published:** 2024-02-27

**Authors:** S. Di Vara, S. Guerrera, D. Menghini, F. Scibelli, E. Lupi, G. Valeri, S. Vicari

**Affiliations:** ^1^Child and Adolescent Neuropsychiatry Unit, Bambino Gesù Children’s Hospital, IRCCS, Rome, Italy; ^2^Department of Life Sciences and Public Health, Catholic University, Rome, Italy

**Keywords:** ASD, sex, psychopathology, cognitive, adaptive functioning

## Abstract

**Introduction:**

Autism Spectrum Disorder (ASD) is a neurodevelopmental disorder characterized by a higher prevalence in male than in female participants. Recent studies have hypothesized the presence of different phenotypes in male and female participants with ASD. The present study aims to assess possible sex differences in cognitive and adaptive functioning, symptomatology of ASD, and psychopathological comorbidities in a large sample of children and adolescents with ASD.

**Methods:**

The study included a total of 2,146 children and adolescents diagnosed with ASD, comprising 1785 boys (mean age 7.12 ± 3.69 years) and 361 girls (mean age 6.25 ± 3.30 years). The age of the participants ranged from 1.35 to 19.05 years (mean age 9.98 ± 3.64). The study sought to include all children and adolescents diagnosed with Autism or ASD.

**Results:**

Present results showed that girls with ASD had lower IQs than boys but similar adaptive functioning. The severity of symptoms of ASD was greater in boys than in girls, as were scores on psychopathological measures. With increasing age, boys with ASD showed greater impairment in social communication skills than girls and increased psychopathological comorbidities. Older girls showed fewer restricted and repetitive behaviors.

**Discussion:**

Exploring phenotypic differences in children and adolescents with ASD fosters an understanding of subtle diagnostic facets that may go unrecognized, allowing for increasingly individualized and tailored interventions.

## 1 Introduction

Autism spectrum disorder (ASD) is a neurodevelopmental disorder characterized by core social impairment, communication difficulties, and the presence of restricted and repetitive interests (American Psychiatric Association, 2013). ASD is diagnosed more often in boys than in girls. An Italian study conducted on a large sample of children with ASD highlighted a prevalence of 1% with a male-to-female ratio close to 3:1 (Narzisi et al., 2020). These findings confirm the results of a systematic review and meta-analysis conducted in the United Kingdom (Loomes et al., 2017), as well as recent American data, which estimated a 3.8-fold higher prevalence among boys than girls and a prevalence estimate of ASD per 1,000 children aged 8 years was 27.6 (Maenner et al., 2023). Previous research has shown a sex ratio of 4:1 across the spectrum, which increases to 8:1 when considering patients with average intelligence (Fombonne, 2003; American Psychiatric Association, 2013; Blumberg et al., 2013; Christensen et al., 2016). Indeed, when examining sex differences in cognitive functioning, it has been observed that the percentage of girls with ASD without intellectual disability is lower compared to boys (Fombonne, 1999, 2009; Nicholas et al., 2008; Frazier et al., 2014; Howe et al., 2015; Mussey et al., 2017) with a male-to-female ratio of 7:1 for high-functioning ASD and 2:1 for individuals with intellectual disability (Fombonne, 2003; Napolitano et al., 2022). Additionally, girls with higher cognitive functioning may exhibit milder symptoms of ASD compared to boys, leading to potential under-recognition and underdiagnosis (Goldman, 2013; Lai et al., 2017a). Other studies have also investigated sex differences in cognitive and adaptive functioning, but the results are mixed (Carter et al., 2007; Lemon et al., 2011; Mandy et al., 2012; Frazier et al., 2014). Similarly, regarding adaptive functioning, there is conflicting evidence. For instance, Frazier et al. (2014) found lower adaptive functioning in female participants with ASD than in male participants, including 304 female participants and 2,114 male participants with ASD from the Simons Simplex Collection, assessed by using multiple instruments for the IQ and the Vineland Adaptive Behavior Scales to evaluate the adaptive behavior. The authors found that reductions in intelligence quotient (IQ) mediated greater social impairment and reduced adaptive behavior in girls with ASD. A similar result was found in the study by Ratto et al. (2018), who had considered a population of 228 children, including 114 girls, assessed by using Wechsler Scales for the IQ and the Vineland Adaptive Behavior Scales to evaluate the adaptive behavior. In contrast, in a recent study conducted by Dellapiazza et al. (2022), a similar clinical profile in adaptive functioning emerged between boys and girls with ASD in a population of 876 children (aged 2 to 16 years) by using Wechsler Scales to assess the IQ and the Vineland Adaptive Behavior Scales to evaluate the adaptive behavior. This result was also confirmed in a recent Italian study (Siracusano et al., 2021). These discrepancies in findings may be attributed to variations in the age range, characteristics, and sample size of individuals with ASD included in the studies, as fewer studies have examined large cohorts of children and adolescents (Szatmari et al., 2012; Frazier et al., 2014; Howe et al., 2015; Knutsen et al., 2019). However, a recent meta-analysis (Saure et al., 2023) demonstrated that the presence of intellectual disability impacts the female ASD phenotype, revealing sex differences in social communication and interaction, sensory processing, language skills, and motor skills. The analysis revealed that girls with ASD and intellectual disability exhibit more severe symptomatology and face greater difficulties compared to boys in these areas.

A part of the sex difference in the prevalence of ASD has been attributed to differences in the symptomatology of ASD. Frazier et al. (2014) conducted a study and found no significant differences in overall indices of ASD symptom severity between the two genders. However, they did observe that social and communication impairment was greater in girls with ASD compared to boys. These findings have been recently confirmed by Dellapiazza et al. (2022).

Due to the prevalence of ASD in boys, the results of many studies have led to a male-oriented classification of ASD (e.g., Bargiela et al., 2016; Hull et al., 2020). As a result, diagnostic tools and criteria are also based on male characteristics, although they are also used in the female population (Tillmann et al., 2018; Navarro-Pardo et al., 2021). The “camouflage hypothesis” (Bargiela et al., 2016; Hull et al., 2020; Napolitano et al., 2022) suggests that girls with ASD may be more likely to be misdiagnosed or experience delays in diagnosis compared to boys with ASD. According to this hypothesis, girls with ASD may exhibit better social abilities (Bargiela et al., 2016; Sedgewick et al., 2016; Hull et al., 2020; Napolitano et al., 2022), fewer restricted and repetitive behaviors (RRB) (Baron-Cohen et al., 2009; Szatmari et al., 2012; Frazier et al., 2014; Lai et al., 2015; Beggiato et al., 2017; Supekar et al., 2017; Knutsen et al., 2019; Napolitano et al., 2022), and more internalizing problems than boys with ASD (Solomon et al., 2012; Hiller et al., 2014; May et al., 2014; Napolitano et al., 2022). However, these issues are still a topic of controversy and the subject of ongoing research. Most of the studies agree that a higher level of restricted and repetitive interests and behaviors occur in boys more than in girls with ASD (Baron-Cohen et al., 2009; Szatmari et al., 2012; Frazier et al., 2014; Lai et al., 2015; Supekar and Menon, 2015; Beggiato et al., 2017; Knutsen et al., 2019). A recent review supports the notion of higher levels of RRB in boys with ASD compared to girls (Napolitano et al., 2022). On the contrary, findings on gender differences in social communication skills were conflicting (Szatmari et al., 2012; Frazier et al., 2014; Van Wijngaarden-Cremers et al., 2014); while some studies showed greater deficits in social development in boys than in girls with ASD (Szatmari et al., 2012; Supekar and Menon, 2015), other studies have shown a similar profile between boys and girls with ASD in terms of social-communicative skills (Szatmari et al., 2012; Van Wijngaarden-Cremers et al., 2014; Lai et al., 2015; Supekar et al., 2017; Stroth et al., 2022; Horwitz et al., 2023). In addition, other studies (Frazier et al., 2014) found that impairment in the social and communication areas was greater in girls with ASD than in boys.

Regarding the presence of psychopathology in boys and girls with ASD, several studies have investigated the relationship between sex and internalizing and externalizing symptoms, revealing higher rates of psychopathological comorbidity in boys than in girls (Guerrera et al., 2019; Lai et al., 2019; Prosperi et al., 2021). Furthermore, previous studies have reported greater externalizing symptoms in boys and greater internalizing symptoms in girls with ASD (Solomon et al., 2012; Hiller et al., 2014). For example, Rødgaard et al. (2021) identified externalizing problems in 16,126 children and adolescents with ASD (age range 0–16) in an interesting registry study conducted on a cohort of 671,103 Danish people. The most frequent comorbidity in boys was ADHD (35% compared to 26% of girls sample), while the most frequent comorbidity in girls was affective disorder (19% compared to 8% of boys sample) and anxiety disorder (19% compared to 10% of boys sample). However, other authors found different results: by using the Children Behavior Checklist (CBCL) (Rescorla, 2005), Frazier et al. (2014) found greater total and externalizing behavior problems, irritability, lethargy, and self-injurious behaviors in girls with ASD than in boys, while Muratori et al. (2019) showed no significant difference between boys and girls.

To date, research on the influence of sex differences on the ASD phenotype has yielded some evidence but some still conflicting results: it is not clear if a difference occurs in the cognitive level and adaptive behavior (generally, intellectual disability was more common in male, but some studies found no difference in cognitive and adaptive level between groups), in the social-communication area (some studies reveal greater impairment in boys, other studies in girls, while other found no difference between groups), and in internalizing and externalizing behavioral problems (most of the studies found more externalizing problems in male and more internalizing problems in female, but some studies found no difference between groups or externalizing problems more severe in girls). These discrepancies could be attributed to methodological differences in studies, such as using different tools, using different samples with limited representation of girls, focusing predominantly on adulthood, and examining limited aspects of the ASD phenotype.

In light of the cited literature data and considering the variability of results regarding potential sex differences in individuals with ASD, this study aims to analyze sex differences within a large sample of children and adolescents diagnosed with ASD. The focus is on intellectual functioning, adaptive functioning, core symptoms of ASD, and associated behavioral and emotional problems. Finally, the study explores the role of age in sex-related phenotypic differences.

## 2 Methods

### 2.1 Participants

The study aimed to include all children and adolescents admitted at the Child and Adolescent Psychiatric Unit from January 2009 to March 2021 with a clinical diagnosis of Autism according to DSM-IV-TR criteria (American Psychiatric Association, 2000) before 2013, or with a clinical diagnosis of ASD according to the DSM-5 criteria (American Psychiatric Association, 2013) if diagnosed after 2013.

A total of 2,146 children and adolescents with ASD were included in the study, consisting of 1785 boys (mean age 7.12 ± 3.69 years) and 361 girls (mean age 6.25 ± 3.30 years). The participants’ ages ranged from 1.35 to 19.05 years (mean age 9.98 ± 3.64). Participants with Social (Pragmatic) Communication Disorder and other Developmental Disorders or psychopathological comorbidities have been excluded. Neuropsychological and psychopathological evaluations were conducted by trained developmental psychologists and psychiatrists to confirm the diagnosis. The diagnoses were further confirmed by using Autism Diagnostic Observation Schedule-Generic (ADOS-G) (Lord et al., 2000) or the revised version of Autism Diagnostic Observation Schedule-2 (ADOS-2) (Lord et al., 2012) administered by a licensed clinician.

The study did not set exclusion criteria based on IQ or language ability. All participants and parents were informed about assessment instruments. Written informed consent was obtained from parents. The study confirmed to the Declaration of Helsinki.

### 2.2 Measures

#### 2.2.1 Cognitive functioning

Cognitive/developmental functioning was assessed through the Wechsler Intelligent Scale for Children IV (WISC-IV) (Wechsler, 2012), Leiter-R (Roid and Miller, 1997) or Leiter-3 (Roid et al., 2013), Raven Progressive Matrices (Raven, 1995), and Griffiths Mental Development Scales-Extended Revised for ages 2–8 (Luiz et al., 2006) according to age, language level, compliance with assessment and timing of the evaluation.

For this study, we considered the full-scale IQ, the non-verbal IQ from Leiter scales and Raven Progressive Matrices (all labeled as IQ), and the General Quotients from Griffiths Mental Development Scales (GQ).

#### 2.2.2 Adaptive functioning

The adaptive functioning was assessed by the parent-report Adaptive Behavior Assessment System—Second Edition (ABAS-II) (Harrison and Oakland, 2003).

When parents were not fluent in Italian, or there were other communication barriers, the semi-structured interview Vineland Adaptive Behavior Scales—Second Edition (VABS-II) (Sparrow et al., 2005) was administered (*n* = 71 children).

Given the high correlation between the domains of the two tools (Harrison and Oakland, 2003), the General Composite score from ABAS-II and the Adaptive Behavior Composite score from VABS-II were considered equivalent measures of adaptive functioning (labeled Adaptive Composite).

#### 2.2.3 ASD symptoms (Clinician’s reports)

The “gold-standard” instruments used in this study to assess ASD symptoms were the Autism Diagnostic Interview-Revised (ADI-R) (Lord et al., 1994), the ADOS-G, and the revised version ADOS-2 (Lord et al., 2012). The ADI-R is a parent-report semi-structured interview; the ADOS is a semi-structured direct assessment of communication, social interaction, and play or imaginative use of materials for individuals with a suspected diagnosis of ASD. Raw Social Affect scores (SA), raw Restricted and Repetitive Behaviors scores (RRB), and total scores were calibrated and transformed into Calibrated Severity Scores (CSS) (Gotham et al., 2009) both for the ADOS-G and ADOS-2 and considered in the statistical analyses.

#### 2.2.4 ASD symptoms (Parents’ reports)

ASD symptoms were also assessed using the Social Responsiveness Scale (SRS) (Constantino et al., 2003) and the Social Communication Questionnaire – Lifetime Version (SCQ) (Rutter et al., 2003). SRS is a widely used parent-report questionnaire that evaluates a child’s social awareness, cognition, communication, motivation, and mannerisms, and higher scores indicate greater social communication difficulties. These scores, together with the total score, were considered for the statistical analyses.

The SCQ – Lifetime version is a 40-item questionnaire with yes/no and a risk cutoff (≥ 15 score), and the total score was considered for the statistical analyses.

#### 2.2.5 Behavioral and emotional symptoms

Behavioral and emotional problems were assessed by the Achenbach System of Empirically Based Assessment (ASEBA) questionnaire (Rescorla, 2005), specifically the Children Behavior Checklist (CBCL) 1.5–5 years and the CBCL 6–18 years questionnaires, reported by parents. Raw scores were converted into t-scores and analyzed in the present study.

According to the cutoff thresholds of Achenbach et al. (2001), *t*-scores >69 were classified as clinically relevant, t-scores between 65 and 69 were classified as borderline, and t-scores <65 indicated non-clinical symptoms. For the internalizing problems, externalizing problems, and total problems scales, t-scores ≥64 were classified as clinically relevant, t-scores between 60 and 63 were classified as borderline, and t-scores <60 indicated non-clinical symptoms.

In the present study, the common scales between the two versions of the questionnaires were considered: anxiety and depression, social withdrawal, somatic complaints, attention problems, aggressive behavior, internalizing, externalizing, and total problems, affective problems, anxiety problems, attentional deficit/hyperactivity problems, and oppositional defiant problems.

A descriptive analysis (Table 1) was conducted for the sociodemographic characteristics of both groups. Differences in age and cognitive ability (IQ/GQ) between the groups (boys and girls with ASD) were assessed using Student’s *t*-test for independent samples. Because of the significant difference between the two groups, we conducted two separate analyses.

**Table 1:** Mean and SD for age, cognitive and adaptive functioning, ASD, emotional and behavioral symptoms by groups.

Demographic characteristics and measures	Male participants			Female participants		
	*N*	*M*	SD	*N*	*M*	SD
Age	1785	7.13	3.69	361	6.26	3.31
**IQ/GQ**						
GMDS-ER 2–8	609	62.62	17.77	169	65.03	20.71
Leiter scales	677	80.93	20.62	119	76.56	20.91
WISC-IV	201	86.76	22.24	26	88.69	26.13
RPM	99	105.03	17.63	15	104.08	22.23
**ASD SYMPTOMS (ADOS/ADOS-2)**						
Social affect CSS	1,454	6.32	1.14	289	6.25	1.67
Restricted and repetitive behaviors CSS	1,454	7.09	1.84	289	6.87	1.81
Total score CSS	1,467	6.39	1.59	291	6.31	1.65
**ASD SYMPTOMS (ADI-r)**						
Scale A	1,056	14.63	5.65	216	14.82	5.72
Scale B	1,056	9.92	4.01	216	9.35	3.68
Scale C	1,057	5.80	2.78	216	5.06	2.78
Scale D	1,051	4.04	1.15	216	4.11	1.03
**ASD SYMPTOMS (SCQ)**						
Total Score	1,009	16.33	8.15	200	15.86	7.72
**ASD SYMPTOMS (SRS)**						
Social awareness	608	67.23	13.70	124	66.94	13.44
Social communication	601	72.79	14.78	123	72.13	15.34
Social cognition	600	78.08	16.80	123	76.24	16.85
Social motivation	602	73.39	16.53	123	73.77	16.29
Autistic mannerisms	601	78.07	19.11	123	77.20	21.20
Total score	799	79.45	17.76	157	79.31	19.24
**ADAPTIVE FUNCTIONING**						
Adaptive composite score (ABAS-II)	1,104	61.54	16.93	227	61.46	17.68
Adaptive composite score (VABS-II)	54	51.74	16.81	17	57.00	19.42
**EMOTIONAL AND BEHAVIORAL SYMPTOMS (CBCL)**						
Anxiety/depression problems	1,333	58.21	8.60	279	56.90	7.74
Somatic complain	1,337	57.24	7.94	280	55.93	6.30
Attention problems	1,336	63.17	8.90	280	63.10	9.74
Aggressive problems	1,338	56.86	7.96	280	55.61	6.75
Internalizing problems	1,438	60.70	10.34	302	58.99	9.98
Externalizing problems	1,438	55.66	9.70	302	54.27	9.03
Total problems	1,438	60.32	10.35	302	58.64	10.22
Affective problems	1,337	60.40	8.95	280	59.14	8.99
Anxiety problems	1,336	59.90	8.71	279	58.13	8.32
Attention deficit/hyperactivity problems	1,337	58.76	7.25	280	58.25	6.95
Oppositional defiant problems	1,335	55.37	6.58	280	54.47	5.59

IQ/GQ, Intelligent Quotient /General Quotient; GMDS- ER 2–8, Griffiths Mental Development Scales-Extended Revised for age 2–8; Leiter scale, Leiter R and Leiter 3; WISC-IV, Wechsler Intelligent Scale for Children – 4th edition; RPM, Raven Progressive Matrices; ADOS, Autism Diagnostic Observation Scale; ADOS-2, Autism Diagnostic Observation Scale – Second Edition; CSS, Calibrated Severity Score; ADI-R, Autistic Diagnostic Interview- revised; ADI-R scale A, Qualitative impairments in reciprocal social Behavior; ADI-R scale B, Qualitative abnormalities in communication; ADI-R scale C, Restricted range of interests and/or stereotypic behaviors; ADI-R scale D, abnormalities development; ABAS-II, Adaptive Behavior Assessment System—Second Edition; VABS-II, Vineland Adaptive Behavior Scales—Second Edition; SCQ, Social Communication Questionnaire; SRS, Social Responsiveness Scale; CBCL, Child Behavior Checklist.

In the first part, comparisons were made between the two groups through multivariate analyses of covariance (MANCOVA) and analyses of covariance (ANCOVA). Potential IQ effects were considered by including participants’ IQ/GQ as a covariate. Post-hoc analyses were performed using Tukey’s HSD test. For all analyses, a *p*-value ≤ 0.05 was considered statistically significant. In addition, for psychopathology, descriptive analyses were performed to explore the distribution of clinical and non-clinical scores within the two groups.

In the second part of the analysis, correlations between age and the different variables considered were investigated. To investigate the relationship between age and clinical measures in both boy and girl groups using IQ/GQ as the control variable, Bonferroni correction was used to correct for multiple comparisons, and the significance level was set at *p* ≤ 0.017 (3 comparisons of CSS), *p* ≤ 0.005 (11 comparisons of CBCL scores), and *p* ≤ 0.008 (6 comparisons of SRS scores).

The statistical software SPSS version 22 (IBM Corporation, Armonk, NY, 2017) was used for analyses.

## 3 Results

### 3.1 Sex and ASD symptoms

Boys and girls with ASD differed in cognitive functioning (IQ/GQ), with higher IQ/GQ (76.13 ± 23.05) of boys than those of girls (72.80 ± 23.47; t_1909_ = 2.37, *p* = 0.02).

A multivariate analysis of covariance (MANCOVA) with group (boys and girls) as between-subject factors and CSS ADOS scores (Social Affect – SA; Repetitive-Restricted Behavior – RRB; Total score) as within-subject factors was run after controlling for IQ/GQ on CSS. A significant main effect of sex (F_1,1,641_ = 6.76, *p* = 0.009, η^2^*p* = 0.004) emerged, with higher CSS scores in boys than in girls. In addition, the main effect of task (F_2,3,282_ = 34.03, *p* < 0.001, η^2^*p* = 0.020) was significant. Post-hoc comparisons (Tukey HSD test) showed higher RRB scores (Mean = 7.06 SD = 1.84) than SA (Mean = 6.30, SD = 1.60, *p* < 0.001) and total score (Mean = 6.39, SD = 1.58, *p* < 0.001). However, SA and total score did not differ (*p* = 0.07). The interaction sex × task was not significant (F_2,3,282_ = 1.08, *p* = 0.35, η^2^*p* < 0.001).

A multivariate analysis of covariance (MANCOVA) with group (boys and girls) as between-subject factors and SRS scores (Social Awareness, Cognition, Communication, Motivation, Mannerisms, and Total score) as within-subject factors was run after controlling for IQ/GQ on SRS sex effect (F_1,675_ = 1.10, *p* = 0.29, η^2^*p* = 0.002) and interaction sex × task (F_5,3,375_ = 0.84, *p* = 0.52, η^2^*p* = 0.001) were not significant in SRS, while task effect was significant (F_1,675_ = 21.08, *p* < 0.001, η^2^*p* = 0.030). Post-hoc comparisons documented an overall higher total score (Mean = 79.37, SD = 17.58) than mannerisms (Mean = 77.87, SD = 19.52, *p* = 0.02), communication (Mean = 77.84, SD = 16.90, *p* = 0.02), motivation (Mean = 73.42, SD = 16.12, *p* < 0.001), cognition (Mean = 72.93, SD = 14.61, *p* < 0.001), and social awareness (Mean = 67.37, SD = 13.66, *p* < 0.001). No differences emerged between mannerisms and communication and between motivation and cognition (*p* always >0.05).

An analysis of covariance (ANCOVA) with group (boys and girls) as between-subject factors and SCQ total scores as within-subject factors was run after controlling for IQ/GQ on SCQ. In addition, SCQ comparisons did not show significant differences (F_1,1,220_ = 2.70, *p* = 0.10, η^2^*p* = 002).

A multivariate analysis of variance (MANOVA) with group (boys and girls) as between-subject factors and ADI-R scales (A, B, C, D) as within-subject factors was run. Results did not show a significant main effect of sex in ADI-R comparisons (F_1,1,261_ = 2.11, *p* = 0.15, η^2^*p* = 0.002). A significant task effect (F_3,3,786_ = 1609.9, *p* < 0.001, η^2^*p* = 0.560) and a significant interaction sex × ADI-R scale (F_3,3,786_ = 3.71, *p* = 0.01, η^2^*p* = 0.002) were found. Post-hoc interaction analyses (Tukey HSD test) of task effect and sex × ADI-R scale did not show significant differences (*p* always >0.05).

### 3.2 Sex and adaptive functioning

An analysis of covariance (ANCOVA) with group (boys and girls) as between-subject factors and Adaptive Composite scores as within-subject factors was run after controlling for IQ/GQ on adaptive functioning. No significant effect was found for sex in the Adaptive Composite score (F_1,1,344_ = 1.24, *p* = 0.27, η^2^*p* < 0.001).

### 3.3 Sex and emotional and behavior symptoms

A multivariate analysis of covariance (MANCOVA) with group (boys and girls) as between-subject factors and CBCL subscales (anxiety/depression problems, somatic complain, attention problems, aggressive problems, internalizing problems, externalizing problems, total problems, affective problems, anxiety problems, attention deficit/hyperactivity problems, and oppositional defiant problems) as within-subject factors was run after controlling for IQ/GQ on CBCL. Results on the CBCL scale showed a significant main effect of sex (F_1,1,518_ = 9.30, *p* = 0.002, η^2^*p* = 0.006), with higher scores in boys than in girls (Mean = 58.64, SD = 0.19 vs. Mean = 57.24, SD = 0.41). In addition, the task effect was significant (F_10,15,180_ = 63.38, *p* < 0.001,η^2^*p* = 0.040) with higher score in attention problems (Mean = 63.01, SD = 9.00) than in internalizing problems (Mean = 60.23, SD = 10.24, *p* < 0.001), affect problems (Mean = 60.05, SD = 8.91), total problems (Mean = 59.76, SD = 10.31), anxiety problems (Mean = 59.42, SD = 8.65; *p* always <0.001), attention deficit/hyperactivity problems (Mean = 58.55, SD = 7.16), anxiety/depression problems (Mean = 57.75, SD = 8.29), somatic complains (Mean = 56.88, SD = 7.64), aggression problems (Mean = 56.44, SD = 7.55), externalizing problems (Mean = 55.16, SD = 9.54), and oppositional-defiant problems (Mean = 55.06, SD = 6.29; *p* always <0.001). Internalizing problems did not differ from total and affective problems, which did not also differ from anxiety problems (*p* always >0.05). In addition, somatic complaints, aggression problems, and externalizing and oppositional-defiant problems did not differ from each other (*p* always >0.05). A significant interaction sex × CBCL scale was found (F_10,15,180_ = 1.93, *p* = 0.04, η^2^*p* = 0.001). Post-hoc analysis on the sex × CBCL scale did not show any significant difference (*p* always >0.05).

Moreover, frequencies of boys and girls with ASD with clinical and non-clinical scores in CBCL scales were compared using *X*^2^ analyses (Table 2).

**Table 2:** Behavioral problems detected by CBCL subscales according to sex.

CBCL subscales	Non-clinical scores		Borderline/clinical score		*X*^2^	*p*
	Male participants	Female participants	Male participants	Female participants		
Anxiety/depression problems	1,035	231	298	48	3.63	0.054
Somatic complain	1,074	240	263	40	4.40	0.036
Attention problems	765	167	571	113	0.54	0.463
Aggressive problems	1,098	255	234	25	12.80	<0.001
Internalizing problems	583	148	855	154	7.34	0.006
Externalizing problems	938	209	500	93	1.76	0.185
Total problems	697	157	741	145	1.23	0.266
Affective problems	928	213	409	66	5.35	0.021
Anxiety problems	864	212	472	67	13.29	<0.001
Attention deficit/hyperactivity problems	1,051	221	286	59	0.01	0.90
Oppositional defiant problems	1,174	264	161	17	8.55	0.003

CBCL, child behavior checklist.

Results showed that the mean scores obtained on each CBCL scale were within a non-clinical range. Only a few subscales were related to internalizing problems (somatic complaints, internalizing problems, affective problems, and anxiety problems) (somatic complaints *X*^2^ = 3.63, df = 1, *p* = 0.036; internalizing problems *X*^2^ = 7.34, df = 1, *p* = 0.006; affective problems *X*^2^ = 5.35, df = 1, *p* = 0.021; anxiety problems *X*^2^ = 13.29, df = 1, *p* < 0.001, respectively). In addition, aggressive and oppositional-defiant problems showed significant sex differences (*X*^2^ = 12.80, df = 1, *p* < 0.001; *X*^2^ = 8.55, df = 1, *p* = 0.003, respectively).

### 3.4 Sex, age, and ASD symptoms

Partial correlations were run with group as independent variables and CSS ADOS scores as dependent variables. After controlling for IQ/GQ and Bonferroni correction (*p* 0.05/3 CSS scores = 0.017), SA was significantly and positively correlated with age and RRB was significantly and negatively correlated to age in boys. In girls, only the total score was significantly and negatively correlated with age. In sum, lower scores in ASD symptoms in girls and lower scores in RRB but higher in SA in boys were associated with older ages (see Table 3).

**Table 3:** Correlation between age and adaptive and between behavioral and emotional measures in relation to sex.

Measures	Male participants		Female participants	
	*r*	*p*	*r*	*p*
**ASD SYMPTOMS (ADOS/ADOS-2)**				
Social affect CSS	0.105	<0.001*	−0.119	0.046
Restricted and repetitive behaviors CSS	−0.072	0.008*	−0.076	0.204
Total score CSS	0.026	0.340	−0.163	0.006*
**ASD SYMPTOMS (SCQ)**				
Total score	0.089	0.007*	−0.101	0.166
**ASD SYMPTOMS (SRS)**				
Social awareness	0.007	0.865	0.018	0.844
Social communication	0.135	0.001*	0.112	0.231
Social cognition	0.117	0.006*	0.162	0.081
Social motivation	0.120	0.004*	0.166	0.075
Autistic mannerisms	0.208	<0.001*	0.166	0.075
Total score	0.141	0.001*	0.132	0.158
**ADAPTIVE FUNCTIONING**				
Composite score	−0.046	0.126	0.009	0.888
**EMOTIONAL AND BEHAVIORAL SYMPTOMS**				
Anxiety/depression problems	0.248	<0.001*	0.144	0.018
Somatic complain	0.137	<0.001*	0.005	0.940
Attention problems	0.084	0.003*	0.106	0.084
Aggressive problems	0.123	<0.001*	0.062	0.310
Internalizing problems	0.097	0.001*	−0.044	0.471
Externalizing problems	0.271	0.339	−0.044	0.476
Total problems	0.153	<0.001*	0.050	0.412
Affective problems	0.236	<0.001*	0.052	0.392
Anxiety problems	0.352	<0.001*	0.270	<0.001*
Attention deficit/hyperactivity problems	0.097	0.001*	0.150	0.014
Oppositional defiant problems	0.102	<0.001*	0.016	0.801

ADOS, Autism Diagnostic Observation Scale; ADOS-2, Autism Diagnostic Observation Scale – Second Edition; CSS, Comparison Score; SCQ, Social Communication Questionnaire; SRS, Social Responsiveness Scale; CBCL, Child Behavior Checklist. *, significative *p*-value after Bonferroni correction.

Partial correlations were run with group as independent variables and SRS scores as dependent variables. After controlling for IQ/GQ and Bonferroni correction (*p* 0.05/6 SRS scores = 0.008), higher SRS scores resulted in a significant correlation with older ages in boys, except for social awareness. No significant correlations between SRS scores and ages were found in girls (see Table 3).

Partial correlations were run with group as independent variables and SCQ score as dependent variables. After controlling for IQ/GQ, higher SCQ scores were significantly correlated with older ages in boys. No significant correlations between SCQ scores and ages were found in girls (see Table 3).

### 3.5 Sex, age, and adaptive functioning

Partial correlations were run with group as independent variables and adaptive composite scores as dependent variables. After controlling for IQ/GQ, adaptive composite scores were not found to correlate with age, both in boys and girls.

### 3.6 Sex, age, and behavioral and emotional symptoms

Partial correlations were run with group as independent variables and CBCL subscales’ scores as dependent variables. After controlling for IQ/GQ and Bonferroni correction (*p* 0.05/11 CBCL scores = 0.005), higher anxiety problem scores were significantly correlated with older ages in girls. In boys, all CBCL scores were significantly and positively correlated with age, except for the externalizing problems scale.

## 4 Discussion

### 4.1 Sex and ASD symptoms

The present study examined differences between boys and girls in a large group of children and adolescents with ASD in cognitive and adaptive functioning, ASD symptoms, and emotional and behavioral problems to improve early diagnosis and individualization of specific treatment.

Present results showed that boys with ASD had greater severity of general symptoms as assessed by the ADOS than girls. This difference was not moderated by intellectual disability or developmental delay, as comparisons were controlled for IQ/GQ. These findings support those from a recent study (Stroth et al., 2022) where boys with ASD had higher impairment than girls. However, sex did not appear to have an effect on any specific ASD symptom, as both boys and girls showed a similar profile with greater impairment in the area of RRB compared to the area of SA and total score obtained. Similarly, no sex differences were found in other measures used to assess ASD symptoms, such as the parent questionnaires SRS and SCQ and the ADI-R interview.

The present results were consistent with previous studies showing no sex differences in the area of social communication (Lai et al., 2015; Stroth et al., 2022; Horwitz et al., 2023), while partially consistent with the literature regarding the absence of sex differences in the area of RRB. Most studies conducted with children and adolescents with ASD had shown greater impairment of RRB in boys with ASD (Van Wijngaarden-Cremers et al., 2014; Napolitano et al., 2022; Saure et al., 2023). However, such findings had not been found in studies conducted with samples of preschool children (Van Wijngaarden-Cremers et al., 2014; Prosperi et al., 2021). Furthermore, RRB in ASD is mediated by the level of cognitive functioning (Bishop et al., 2006; Richler et al., 2010; Prosperi et al., 2021), and thus, the sex differences in RRB found in previous studies were likely to be due to intellectual disability rather than ASD.

An explanation of the current results could be linked to having control for IQ/GS. In fact, this might have had an effect on the non-recognition of RRB and influenced the sample size by including a larger number of preschool participants (Lai et al., 2019).

In addition, retrospective parent reports of ASD symptoms may differ substantially from clinicians’ direct observational assessments, particularly when ASD symptoms are assessed by questionnaires or interviews such as the ADI-R or SCQ. These instruments ask parents to report behaviors exhibited by their children many years earlier, and as a result, there can be discrepancies between parental ratings and direct observations of behavior. This conclusion was supported by previous research suggesting that parental ratings and direct observation of behavior did not always agree (Winsler and Wallace, 2002; Voigt et al., 2007; Hartley and Sikora, 2009).

### 4.2 Sex and adaptive functioning

The results support data from recent studies in which sex differences did not affect the adaptive function of boys and girls with ASD (Siracusano et al., 2021; Dellapiazza et al., 2022). However, previous studies reported lower adaptive functioning in girls with ASD than in boys (Frazier et al., 2014; Howe et al., 2015; Ratto et al., 2018). The heterogeneity in sample composition and characteristics of the participants could explain the difference in the results. For example, the group of participants with ASD in this study included a wider age range than that considered by Ratto et al. (2018), who included participants aged 6 to 16 years. Nevertheless, the participants in the study by Howe et al. (2015) were recruited from four different datasets, and the authors stated that sex differences in adaptive functioning emerged in only two of the four datasets, suggesting that such differences in results may depend on the specifics of the sample considered.

### 4.3 Sex and behavioral and emotional symptoms

In the present study, the scores obtained on the different scales of the CBCL did not reach clinically relevant levels in most cases, except for internalizing problems. However, the results showed that the overall scores of the boys were higher than those of the girls, highlighting a difference between the groups. In preschoolers, Prosperi et al. (2021) showed a higher prevalence of emotional and behavioral symptoms in boys than in girls, as assessed by CBCL. Similarly, Guerrera et al. (2019) found that boys had more internalizing problems than girls in a large sample of children and adolescents with ASD. On the other hand, the high presence of externalizing problems in boys with ASD was also supported by findings from other studies (Solomon et al., 2012; Frazier et al., 2014; Hiller et al., 2014; May et al., 2014). While the current results from the male group with ASD were consistent with findings from previous studies, the present findings from female groups contrasted with previous large-sample studies that found higher psychopathological symptoms in girls (Frazier et al., 2014; Napolitano et al., 2022). As hypothesized in a recent review by Lai et al. (2019), the heterogeneity in the results could be explained by differences in group composition (age, sex, and IQ), country of origin, or year of publication of the studies analyzed. Another consideration for the discrepancies between studies on the prevalence of psychopathology in ASD is the difficulty in determining whether psychopathological symptoms are truly part of the ASD phenotype or part of a comorbid psychiatric disorder.

### 4.4 Sex, age, and ASD symptoms

Results showed that increasing age was associated with a reduction in total ASD symptoms in girls, while boys showed a reduction in RRB and greater social-communicative skills. The association between total ASD symptoms and age in girls appeared to be consistent with the well-documented trend of later diagnosis in girls with ASD than boys. Girls with ASD, particularly those with mild symptoms or adequate cognitive functioning, were often identified or diagnosed later than boys (Giarelli et al., 2010; Begeer et al., 2013). A recent review (Lai and Szatmari, 2020), in explaining the reasons for the late diagnosis of ASD in girls, hypothesized that “camouflage” may be related to a late onset of socio-communicative and behavioral difficulties (Sedgewick et al., 2016). This may have led to the recruitment of girls and adolescents with ASD at an older age than boys in sex difference studies, explaining the often-conflicting results.

Regarding RRB, recent studies showed that more RRB was present in adolescent boys than in younger groups of male children with ASD (Van Wijngaarden-Cremers et al., 2014; Stroth et al., 2022). However, the current findings supported a recent longitudinal study showing a reduction in RRB with increasing age (Courchesne et al., 2021). Specifically, using the ADI-R, Courchesne et al. (2021) studied a population of 206 children at aged 6 and 8 years, respectively. They found a decreasing trend in RRB with increasing age, except for noise sensitivity and circumscribed interests.

Finally, results in the socio-communicative domain assessed by parent-report questionnaires (i.e., SCQ and SRS) partially support the above findings. Less impairment in social behavior in older girls than in older boys (consistent with direct observation findings with the ADOS) and reduced social functioning in boys were found. The discrepancy between direct and indirect observation of ASD symptomatology is not new, as it has already been addressed in the literature (Sedgewick et al., 2016; Lai et al., 2017a), and further studies would be needed to investigate this issue.

### 4.5 Sex, age, and behavioral and emotional symptoms

Regarding the correlations between age and behavioral and emotional symptoms, the present results showed an increase in all CBCL scores with increasing age in boys, whereas girls only showed greater anxiety symptoms at older ages. Some studies showed an increase in anxiety symptoms, including social avoidance, negative appraisal, and inhibition with older age (Gadow et al., 2004, 2005; Kuusikko et al., 2008), particularly in girls, with a greater increase in symptoms during adolescence (Gotham et al., 2015; Napolitano et al., 2022). A recent review (Napolitano et al., 2022) reported sex differences in the age trajectory of psychopathological comorbidities: in early adolescence, results from parent-report questionnaires showed greater depressive symptoms in girls with ASD than in boys. In late adolescence, ASD boys and girls showed similar levels of depressive symptoms, although boys with ASD appeared to have an increase in symptoms over time, which is partially consistent with our findings. Finally, with regard to anxiety, the review highlighted higher levels of anxiety in girls with ASD than in boys in early adolescence.

However, other studies have not reported a consistent relationship between age and anxiety (Sukhodolsky et al., 2008; White and Roberson-Nay, 2009). In 2008, Simonoff et al. (2008) suggested that the diagnostic overshadowing underlies the underestimation of psychiatric comorbidities in neurodevelopmental disorders. In fact, Davis et al. (2010) proposed these differences may be due to a different focus and interpretation of anxiety behaviors, which are often assessed as characteristic of ASD but are qualitatively different from the social impairments of individuals with ASD.

Finally, studying sex differences in individuals with ASD, we cannot fail to consider that recently, attention has been focused on neuroanatomical sex differences, as previously emphasized by Lai et al. (2017b). However, as highlighted, studies in this area are still limited and often underpowered due to the low number of girls in the samples studied. Indeed, Ecker et al. (2017) reported lower cortical thickness (CT) in ASD girls but greater CT in ASD boys, while more recent authors did not find differences (Van Rooij et al., 2018). Finally, Bedford et al. (2020), despite not finding significant sex differences, identified a sex-specific relationship between the severity of ASD symptoms and CT, explaining the varied literature results based on the number of girls present in the samples of different studies.

## 5 Strengths and limitations

In our view, the present study, examining a sample of 2,146 children and adolescents, including 1785 boys and 361 girls, with a wide age range (from 1.35 to 19.05 years), provides a detailed insight into phenotypic characteristics associated with the diagnosis of ASD. In contrast to some previous literature studies, we do not find sex differences concerning symptomatology and adaptive functioning. On the contrary, we find sex differences in the associated behavioral and emotional symptoms, with higher scores of internalizing problems in boys compared to girls, confirming the results of some literature studies that considered a population with a smaller age range (Guerrera et al., 2019) or focused on a preschool population (Prosperi et al., 2021). Indeed, according to our results, age would play a significant role in mediating sex differences related to both core ASD symptomatology and associated behavioral and emotional symptoms, with boys with ASD with increasing age showed greater impairment in social-communication skills and major behavioral and emotional symptoms than girls, and girls with increasing age had reduced amounts of RRB and only showed greater anxiety symptoms than boys.

One of the strengths of the present study is the large size of the sample, which allows for a comprehensive examination of sex differences in children and adolescents with ASD. Furthermore, few studies have utilized CSS for specific domains of the ADOS-2 (Lord et al., 2012). By utilizing the CSS, this study can compare different versions and modules of the ADOS and provides a measure of ASD symptoms independent of age and level of language development. This makes the CSS more suitable than raw ADOS scores for assessing the severity of ASD (Gotham et al., 2009). Moreover, compared with the present study, most research has examined specific emotional and behavioral problems, focusing exclusively on the macro-category of internalizing and externalizing symptoms.

This study also has limitations that need to be pointed out. First, the heterogeneity of participants in terms of age and cognitive functioning poses a challenge. The wide range of age and IQ/GQ included in the study allows for a representative picture of a pediatric population with ASD. However, it also makes it difficult to directly compare our results with previous studies, as our participants had an age range not typically included in the existing literature. Similarly, few studies have examined the combined effects of age and sex on psychiatric comorbidities in children and adolescents with ASD. As a result, it is difficult to compare the current results with existing data. Furthermore, the clinical assessments of ASD symptoms of the present study were conducted over an extended period using different tools, such as the ADOS-G and the revised version of the ADOS-2. Consequently, it had to include raw total scores for ADOS-G and CSS for ADOS-2, which might have introduced some variability in the data. Moreover, the retrospective nature of this study provides only a snapshot of the population’s characteristics, offering valuable preliminary data. However, relying predominantly on cross-sectional data can make it difficult to identify specific features and changes in ASD concerning different age and sex groups (Kaat et al., 2021). Future research should focus on studying sex differences over time with longitudinal cohort studies and groups with ASD matched for cognitive functioning. Future longitudinal studies focusing on age and sex differences, along with their association with comorbid psychiatric conditions, may offer valuable insights into the evolution of symptoms and help develop more targeted interventions. Additionally, conducting surveys of at-risk populations, such as siblings, could contribute to a better understanding of the relationship between ASD characteristics and sex.

## 6 Conclusion

The present study explored individual differences in ASD phenotype and comorbid psychiatric conditions manifest in a large pediatric sample. Results showed that the cognitive functioning of girls is lower than that of boys. With increasing age, boys with ASD showed greater impairment in social-communication skills than girls, and girls had reduced amounts of RRB. The adaptive functioning was unrelated to sex, whereas differences in emotional and behavioral problems seemed to emerge with increasing age, especially in boys.

The National Institute for Health and Care Excellence (NICE) guidelines on the treatment of children and adolescents with ASD focus on the need for increasingly individualized treatments aimed at promoting a better quality of life for the individual. To indicate individualized treatment, clinicians need to be aware of individual characteristics mediated by variables such as sex and age, which, in turn, can influence the expression of the complex clinical picture. Therefore, this study, analyzing possible sex differences in a large sample of the population of children and adolescents with ASD, aims to contribute to the understanding of diagnostic characteristics of ASD in children and adolescents and, consequently, to the possibility of early diagnostic recognition and, secondarily, the initiation of specific treatment. Understanding sex differences in ASD can lead to early interventions for diagnosis and therapy, and it may provide valuable insights to improve diagnostic accuracy, especially for girls with ASD who may be underdiagnosed due to the “camouflage” phenomenon.

## Data availability statement

The raw data supporting the conclusions of this article will be made available by the authors, without undue reservation.

## Ethics statement

The studies involving humans were approved by the local Ethics Committee (protocol code: 2423_OPBG_2021), approved on October 27, 2021. The studies were conducted in accordance with the local legislation and institutional requirements. Written informed consent for participation in this study was provided by the participants’ legal guardians/next of kin.

## Author contributions

SVa: Conceptualization, Data curation, Formal analysis, Methodology, Writing – original draft, Writing – review & editing. SG: Conceptualization, Writing – original draft, Writing – review & editing. DM: Conceptualization, Formal analysis, Methodology, Supervision, Writing – review & editing. FS: Conceptualization, Data curation, Writing – original draft. EL: Conceptualization, Data curation, Writing – original draft. GV: Conceptualization, Methodology, Supervision, Writing – review & editing. SVi: Conceptualization, Methodology, Supervision, Writing – review & editing.
